# Macrophages regulate the clearance of living cells by calreticulin

**DOI:** 10.1038/s41467-018-06807-9

**Published:** 2018-11-07

**Authors:** Dmitri V. Krysko, Kodi S. Ravichandran, Peter Vandenabeele

**Affiliations:** 1 Department of Basic Medical Sciences, Ghent University, Ghent, 9000 Belgium; 2 National Research Lobachevsky State University of Nizhni Novgorod, Nizhny Novgorod, 603950 Russia; 3 The Center for Cell Clearance and the Department of Microbiology, Immunology, and Cancer Biology, University of Virginia, Charlottesville, 22908-0734 VA USA; 4 Inflammation Research Center, VIB, Ghent, 9052 Belgium; 5 Department of Biomedical Molecular Biology, Ghent University, Ghent, 9052 Belgium; 6 Methusalem Program, Ghent University, Ghent, 9052 Belgium

## Introduction

**Fig. 1:**
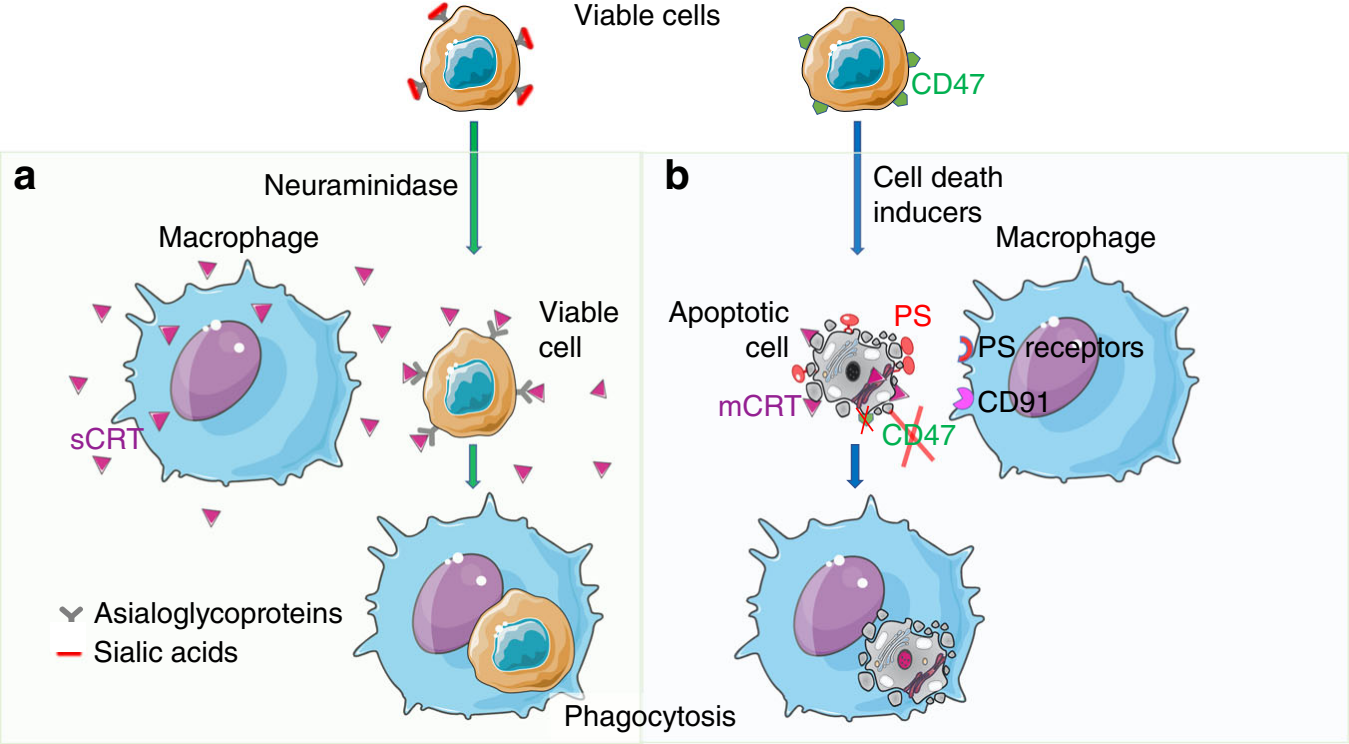
Mechanisms of recognition of viable (**a**) and apoptotic (**b**) cells. **a** A soluble calreticulin (sCRT) secreted by activated macrophages is capable of binding to asialoglycans on viable target cells, depending on their developmental or damage state, which in turn, marks them for uptake. Neuraminidase-4 is one enzyme that could be involved in the generation of cell surface asialoglycans that bind sCRT. The cell-surface mCRT on the target cells can now interact with CD91, which is also known as low-density lipoprotein receptor-related protein 1 (LRP1). **b** CRT and CD91 have also been linked to immunogenic apoptotic cell death. On the surface of apoptotic cells expose phosphatidylserine (PS) and mCRT that function as “eat me” signals. The mCRT expression is particularly linked to cell death induced by agents that are known to induce immunogenic forms of apoptotic cell death. The expression of “don’t eat me” signals (e.g., CD47) on the apoptotic cells is either reduced or its location modified. Many membrane-bound receptors for PS have been reported, including those that bind PS directly or indirectly via bridging molecules. mCRT can bind CD91 on the phagocyte. The authors thank Servier for the figures that were produced using Servier Medical Art (www.servier.com)

Recently, Feng, Weissman and colleagues demonstrated that activated macrophages secrete calreticulin (CRT), which binds to the surface of viable target cells and marks them for removal by programmed cell phagocytosis (PrCP)^1^. CRT is one of the ‘eat me’ signals, which is often thought to localize in the lumen of the endoplasmic reticulum (ER), but was also found on the plasma membrane of apoptotic cancerous cells (Fig. 1), where it is believed to function as an ‘eat me’ signal^2^ and a damage-associated molecular pattern (DAMP) responsible for the immunogenicity of apoptotic cancerous cells^3^. Remarkably, some viable cells also expose CRT but are apparently protected from engulfment. One of the mechanisms for this protection is related to repelling signals mediated by so-called ‘don’t eat me’ signals concurrently expressed on the cells, such as CD31, CD200 and CD47^4^. Importantly, clearance of viable cells has been recently recognized in a few contexts, and it has been intriguing, in part due to the lack of an obvious mechanism, and also for its relevance. Some examples of reported live cell clearance include suicidal emperipolesis^5^, entosis^6^, phagoptosis^7^, and cell cannibalism^8^. However, the source and specific role of CRT in the engulfment of viable cells and the binding moiety on the surface of viable cells have not been known yet.

Feng et al. showed that activated macrophages are a source of CRT bound on the surface of neutrophils. In order to distinguish between efferocytosis^9^ of apoptotic neutrophils versus the uptake of CRT-marked ‘live’ or ‘aged’ neutrophils by PrCP, the authors performed a comparison between wild type and Bcl-2-overexpressing neutrophils and confirmed a 25-old report from the same group that the eventual extent of uptake is not affected by blocking apoptosis in the target cells^10^. However, although several mechanisms of CRT exposure on the surface of cells undergoing immunogenic apoptosis have been described^11^, which mechanisms of CRT secretion are used by macrophages to enhance PrCP are currently unknown and many more interesting and challenging findings are expected. In this regard it has also been suggested the links between ER calcium depletion and detection of ER-resident proteins, including CRT, in the extracellular space^12^.

What does the CRT bind to on the surface of neutrophils and possibly other viable cells engulfed via PrCP? Since CRT is involved in the quality control of glycoproteins in the ER by a lectin-like function, the authors asked whether during PrCP, the extracellular binding of CRT on neutrophils (and other living cells) may also involve sugar moieties. Feng et al. found that CRT selectively bound to asialoglycans displaying the Tri-antennary and multivalent type II chain epitopes (Tri/m-II). This specificity of CRT-binding was confirmed on mature murine neutrophils, as well as on the cell surface of a viable human acute myeloid leukemia cell line (HL60) and a human colon cancer cell line (SW620) by competitive binding with Phytohaemagglutinin-L (PHA-L), a lectin with the same specificity for Tri/m-II^1^. These findings demonstrate that this CRT binding and specificity can be extended to other cell types than neutrophils and suggest that PrCP could be a general phenomenon that may occur during different biological processes such as inflammation resolution and anti-cancer surveillance (Fig. 1).

Neuraminidase is a glycoside hydrolase that removes sialic acids from the terminal positions of glycans and exposes the cryptic Tri/m-II, leading to increased CRT binding. Having identified the asialoglycan epitope involved in CRT binding, the authors then examined whether CRT-binding and PrCP could be modulated by neuraminidase treatment^1^. They found that this treatment significantly promoted PrCP of different types of viable human cancer cells by both human and mouse macrophages. In a complementary approach, Feng et al. found that knocking out the neuraminidase-4 gene (neu-4) in a cancer cell line, significantly decreased PrCP. To test the potential usefulness of these observations to clinical settings, the authors mined transcriptome databases of cancers and this revealed higher expression of genes promoting the removal of sialic acids (such as neuraminidases) correlated with an improved survival, while higher expression of the genes enhancing sialic acid expression (such as sialyl transferases) correlated with a worse outcome. Also, during normal and pathological haematopoiesis the level of CRT or PHA-L binding correlated inversely with cellular half-life, with highest binding in blast cells and lowest in multipotent progenitors, leukemia stem cells and haematopoeitic stem cells. These studies indicate that regulation of asialoglycan levels on the cell surface and the subsequent PrCP due to the CRT mediated recognition could be important during homeostasis and inflammation resolution and as well as immunosurveilance.

This work by Feng et al. reveals several new pieces of information and as all good studies, also raises a number of questions. First, previous studies have suggested that CRT on the surface of apoptotic cells functions as a (DAMP responsible for the immunogenicity of apoptotic cancerous cells^3,13,14^. This current work places CRT in a new context, i.e. in the clearance of ‘viable’ cancer cells with CRT acting as an ‘eat me’ signal. Whether modulation of the asialoglycan epitope by neuraminidase treatment also modulate the uptake of apoptotic cells, whether this will modulate their immunogenicity is a question that could also be relevant for tumor immunotherapy. In this regard, Feng et al. showed that neuraminidase treatment leads to a decrease in tumor engraftment and growth in mice, and that in humans, stronger expression of genes enhancing sialic acids expression correlates with a worse outcome^1^. Future studies addressing the anti-tumor responses could be useful. This raises also the question whether other phagocytic cells, such dendritic cells, can secrete CRT to decorate their preys, and whether engulfment of viable cancer cells would lead to antigen presentation. Second, the current work suggesting the modulation of asialoglycoproteins on the surface of viable cells and the section of CRT from macrophages, begin to provide mechanistic insights. Although not focus of this work, whether different types of tissue macrophages as well as their differentiation (such as M1-like versus M2-like signatures) might alter CRT secretion, or whether the PrCP is rather a feature of tumor associated macrophages remain to be seen. Finally, beyond tumor immunosurveillance, an intriguing observation is the interaction between viral neurominidases (such as Influenza virus) and the host cells. Indeed Influenza neurominidases have been shown to enhance phagocytosis of Influenza-infected cells through desialylation^15^. Future investigations will lead to a better understanding of the molecular mechanisms of the clearance of viable cells during cancer and viral infections with relevance to anti-tumor therapies as well as normal hematopoietic development.
